# From heart to brain: a Case Report of individualized antithrombotic management for left ventricular thrombus and stroke in a young patient with acute myocardial infarction

**DOI:** 10.3389/fphar.2026.1829968

**Published:** 2026-06-11

**Authors:** Jincheng Wang, Xi Zhang, Qing Jin, Qiu Chen, Fuding Guo, Qiang Xue

**Affiliations:** 1 Department of Cardiology, The Affiliated Yan’an Hospital of Kunming Medical University, Kunming, Yunnan, China; 2 Key Laboratory of Cardiovascular Disease of Yunnan Province, Kunming, Yunnan, China; 3 Clinical Medicine Center for Cardiovascular Disease of Yunnan Province, Kunming, China

**Keywords:** antithrombotic therapy, case report, left ventricular thrombus, myocardial infarction, stroke

## Abstract

**Background:**

Left ventricular thrombus (LVT) is a serious complication of acute myocardial infarction (AMI), conferring a high risk of systemic embolism. The management of LVT becomes exceedingly challenging when complicated by acute ischemic stroke and recent percutaneous coronary intervention (PCI), creating a therapeutic trilemma that lacks guideline-directed recommendations.

**Case summary:**

A 27-year-old male with hypertension and diabetes presented with acute anterior ST-segment elevation myocardial infarction. Due to unsuccessful initial revascularization at a local hospital, he underwent delayed PCI on day 10 post-infarction at our center. On the first post-PCI day, he developed acute ischemic stroke, and echocardiography confirmed a mobile left ventricular thrombus as the embolic source. Navigating competing risks of stent thrombosis, hemorrhagic transformation, and recurrent embolism, an individualized six-phase antithrombotic strategy was implemented. This dynamic approach included acute-phase de-escalation, sequential anticoagulation with rivaroxaban followed by warfarin guided by thrombus response, stepwise de-escalation after complete revascularization, and eventual transition to long-term dual-pathway inhibition. Complete LVT resolution was achieved within 6 months, and the patient’s neurological deficits fully recovered. No recurrent LVT, ischemic events, or major bleeding occurred during 18 months of follow-up.

**Conclusion:**

This case highlights the complex management challenges of left ventricular thrombus and acute stroke following myocardial infarction, and underscores the value of individualized, phased antithrombotic strategies guided by dynamic risk assessment and serial imaging, rather than rigid adherence to single-disease guidelines.

## 1 Introduction

Left ventricular thrombus (LVT) is a major complication of acute myocardial infarction (AMI), potentially leading to severe embolic events such as stroke (McCarthy et al., 2018). Although its incidence has declined with widespread use of percutaneous coronary intervention (PCI) and dual antiplatelet therapy (DAPT), systemic embolism remains a critical clinical concern. A recent study reported that during five-year follow-up after myocardial infarction, patients with LVT had a 16.3% risk of systemic embolism, a 5.5-fold increase over those without LVT (Maniwa et al., 2018). LVT also nearly doubles long-term mortality (McCarthy et al., 2019). Importantly, certain high-risk populations—such as those with large anterior infarctions, delayed revascularization, or prothrombotic conditions like metabolic syndrome—may face disproportionately elevated thrombotic risk, irrespective of age (Kok et al., 2014).

The simultaneous occurrence of AMI and acute ischemic stroke (AIS) is exceedingly rare but challenging (Obaid et al., 2019). For patients with AMI undergoing PCI who develop LVT and subsequent stroke, management becomes a therapeutic trilemma. Clinicians must simultaneously balance three competing risks: (1) recurrent systemic embolism from LVT, necessitating effective anticoagulation; (2) stent thrombosis, requiring potent antiplatelet therapy; and (3) hemorrhagic transformation, requiring caution with antithrombotic agents. Current guidelines recommend anticoagulation for isolated LVT and DAPT after PCI (Ibanez et al., 2018; O'Gara et al., 2013), but provide no specific advice for this triple coexistence.

We report a 27-year-old male with metabolic syndrome who developed LVT and cerebral embolism shortly after delayed PCI for anterior ST-segment elevation myocardial infarction (STEMI). The report details an individualized, phased and dynamically adjusted antithrombotic strategy that successfully achieved thrombus resolution, with no recurrences or ischemic/bleeding complications observed during an 18-month follow-up period. This case offers a reference for managing cardiovascular-cerebrovascular overlap syndromes, emphasizing that dynamic risk adjustment, rather than rigid adherence to single-disease guidelines, may be key to favourable outcomes.

## 2 Case presentation

### 2.1 Patient presentation and initial management

A 27-year-old male (BMI 28 kg/m^2^) with a history of hypertension and type 2 diabetes mellitus presented to a local hospital on 25 November 2024, with 6 h of severe chest pain. There was no family history of premature cardiovascular disease or hypercholesterolemia. Electrocardiography confirmed acute anterior ST-segment elevation myocardial infarction. Emergency coronary angiography revealed total occlusion of the proximal left anterior descending artery (LAD) and severe stenosis of the distal right coronary artery(RCA). The initial PCI was unsuccessful. The patient was medically managed with dual antiplatelet therapy (DAPT: aspirin and ticagrelor) and guideline-directed medications. Due to persistent symptoms, he was transferred to our tertiary center on 2 December 2024.

### 2.2 Pre-procedural evaluation and risk stratification

**FIGURE 1:**
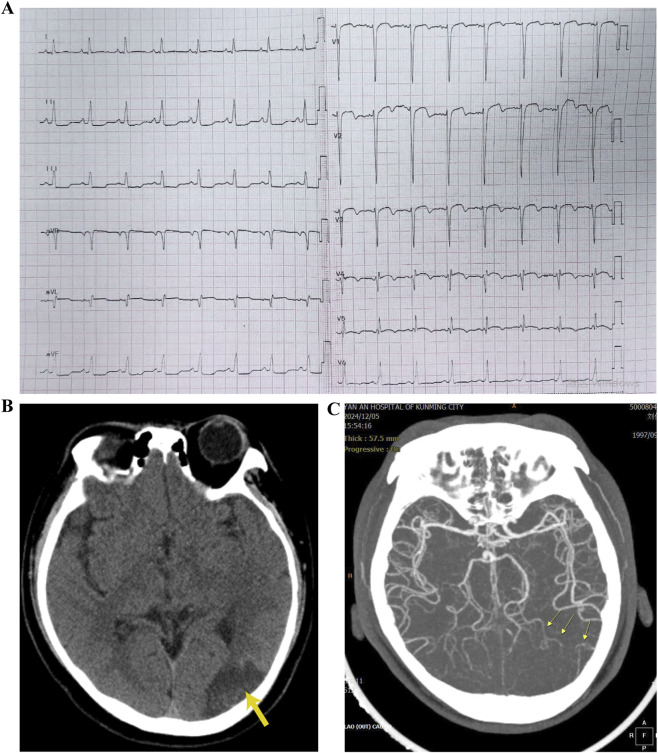
Electrocardiographic and neuroimaging findings. **(A)** Twelve-lead electrocardiogram on admission showed sinus rhythm, ST-segment elevation in leads V1-V4, poor R-wave progression in chest leads, and ST-segment depression in inferior leads. **(B)** Emergency brain CT on the first post-PCI day showing a wedge-shaped hypodense lesion in the left occipital lobe (3.5 × 3.1 cm). **(C)** CT angiography showing mild-to-moderate stenosis of the left posterior cerebral artery (P2 segment, arrow), with no large vessel occlusion.

**FIGURE 2:**
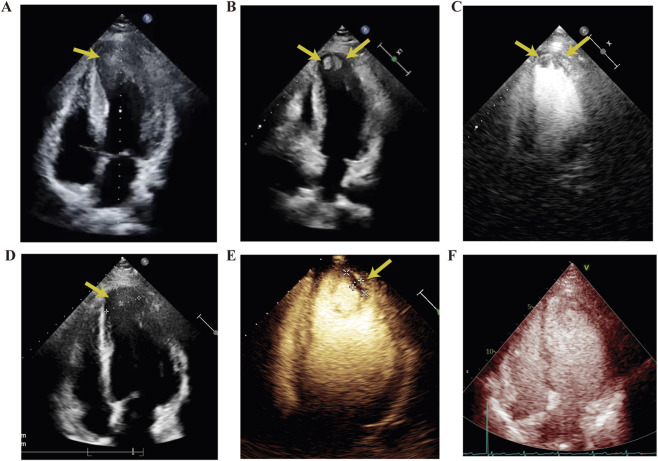
Serial echocardiographic monitoring of left ventricular thrombus. **(A)** Pre-PCI transthoracic echocardiography showing apical hypokinesia with vortex-like echoes indicating blood stasis. **(B)** Echocardiography immediately after stroke onset revealed two mobile mass-like thrombi in the left ventricular apex (1.6 × 1.1 cm and 1.3 × 1.1 cm, arrows). **(C)** One-week follow-up contrast-enhanced TTE showing persistent apical thrombus despite rivaroxaban therapy (arrow). **(D)** Routine TTE at 1 month revealing an apical ventricular aneurysm (3.0 × 1.5 cm, arrow) without significant thrombus. **(E)** Contrast-enhanced TTE at 1 month demonstrating residual thrombus (1.8 × 0.7 cm, arrow) with reduced size and mobility. **(F)** Six-month contrast-enhanced TTE confirming complete resolution of left ventricular thrombus.

Upon admission, vital signs were stable. Physical examination revealed moist rales in the lower lung fields bilaterally. Electrocardiogram showed sinus rhythm, ST-segment elevation in leads V1-V4, poor R-wave progression in chest leads, and ST-segment depression in inferior leads (Figure 1A). Laboratory tests demonstrated markedly elevated cardiac and metabolic markers: cardiac troponin I 1.7 μg/L; NT-proBNP 2,226 ng/L; glycated hemoglobin 11.8%; total cholesterol 9.36 mmol/L; triglycerides 3.82 mmol/L; low-density lipoprotein cholesterol 7.15 mmol/L; and uric acid 626 μmol/L. Hypercoagulability work-up (antiphospholipid antibodies, coagulation function and antinuclear antibodies) showed no abnormalities. Transthoracic echocardiography (TTE) showed anterior/apical hypokinesia with LVEF 46%. No thrombus was seen, but vortex-like echoes indicating blood stasis were observed in the apical region (Figure 2A). Preprocedural risk scores: GRACE 127 (intermediate ischemic risk), CRUSADE 30 (low in-hospital bleeding risk).

### 2.3 Revascularization and early postoperative course

**FIGURE 3:**
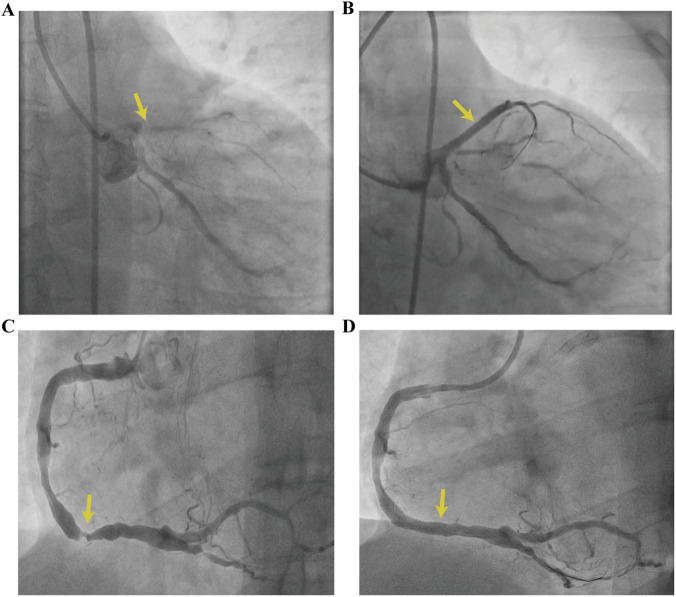
Coronary angiography and percutaneous coronary intervention. **(A)** Pre-PCI angiography showing complete occlusion of the proximal left anterior descending artery (LAD, arrow). **(B)** Post-PCI angiography demonstrating successful LAD revascularization with two drug-eluting stents (arrow) and TIMI 3 flow. **(C)** Right coronary angiography showing 95% stenosis in the distal segment and posterior descending artery (arrow). **(D)** One month later, elective PCI of the right coronary artery achieved complete revascularisation (arrow).

Given the risk of persistent ischemia and the need to improve ventricular remodeling, coronary intervention was performed on day 10 post-infarction (4 December 2024). Repeat angiography confirmed proximal LAD occlusion (Figure 3A) and 95% distal RCA/posterior descending artery (PDA) stenosis (Figure 3C). Under intravascular ultrasound guidance, two drug-eluting stents were implanted in the LAD (Figure 3B). Due to transient hemodynamic instability, revascularization of the RCA was deferred as an elective procedure. To mitigate perioperative thrombotic risk, therapeutic enoxaparin sodium (4000 IU q12) was added to DAPT post-PCI.

### 2.4 Acute stroke and diagnosis of left ventricular thrombus

On the first post-PCI day, the patient acutely developed right-sided homonymous hemianopia and right limb weakness (muscle strength grade 3/5), with a National Institutes of Health Stroke Scale (NIHSS) score of 8. Emergency brain CT showed a wedge-shaped left occipital hypodensity (3.5 × 3.1 cm), consistent with acute ischemic stroke (Figure 1B). The Posterior Circulation Alberta Stroke Program Early CT Score (PC-ASPECTS) was 9, suggesting moderate ischemic change. The initial SITS-SICH score was 8, indicating moderate hemorrhagic transformation risk (6–8 points). CT angiography revealed only mild-to-moderate left posterior cerebral artery (P2) stenosis, no large vessel occlusion (Figure 1C). Consequently, mechanical thrombectomy was not indicated. Intravenous thrombolysis was contraindicated because of recent PCI and concurrent DAPT/enoxaparin (Berge et al., 2021). Immediate echocardiography demonstrated two mobile, mass-like thrombus (measuring 1.6 × 1.1 cm and 1.3 × 1.1 cm) in the left ventricular apex (Figure 2B), confirming LVT as the embolic source.

### 2.5 Individualized, staged antithrombotic strategy

Following cerebral infarction, comprehensive risk reassessment guided long-term management. The SYNTAX score was 33.5 (high-risk coronary anatomy) and the HAS-BLED score was 3 (high bleeding risk). Navigating this dual high-risk profile, a dynamic six-phase antithrombotic strategy was implemented:

Phase 1 (Acute ischemic stroke): Given the imminent risk of hemorrhagic transformation, enoxaparin was stopped. DAPT was de-escalated to indobufen plus clopidogrel to prioritize intracranial safety. Neuroprotective agents (edaravone and butylphthalide injection) were also administered.

Phase 2 (Reinitiation of anticoagulation): On the second post-PCI day, the patient’s neurological deficit of hemianopia improved, with limb muscle strength recovering to grade 4/5, the NIHSS score decreased from 8 to 3, the SITS-SICH score decreased from 8 to 4, and repeated CT excluded intracranial hemorrhage. With stable bleeding indices, the primary threats shifted to recurrent left ventricular thromboembolism and in-stent thrombosis. Therefore, rivaroxaban (15 mg once daily) was added to DAPT, establishing short-term triple therapy; a proton pump inhibitor was co-administered to reduce gastrointestinal bleeding risk.

Phase 3 (Switch to warfarin): After 7 days of treatment, the patient had fully recovered neurologically (NIHSS 0). Contrast-enhanced echocardiography showed no reduction in thrombus size or mobility compared with the initial post-stroke study (Figure 2C). Given the persistently high embolic risk (mobile LVT, heart failure, diabetes, metabolic syndrome) and a reduced bleeding risk profile, we switched anticoagulation to warfarin (target INR 2.0–2.5), while continuing DAPT (indobufen + clopidogrel) to achieve a monitorable, dose-adjustable regimen.

Phase 4 (De-escalation after complete revascularization): One-month routine echocardiography revealed a stable apical aneurysm (3.0 × 1.5 cm) with no obvious thrombus (Figure 2D), whereas contrast-enhanced echocardiography showed residual LVT (1.8 × 0.7 cm) with reduced size and mobility (Figure 2E). After confirming LAD stent patency and successfully completing elective PCI of the RCA lesion (Figure 3D), the ischemic risk from the coronary lesion was substantially reduced. Accordingly, triple therapy was de-escalated to dual therapy (warfarin plus clopidogrel).

Phase 5 (Post-thrombosis antiplatelet therapy): Six-month contrast-enhanced echocardiography confirmed complete LVT resolution (Figure 2F). Warfarin was discontinued, and therapy was transitioned to indobufen plus clopidogrel for secondary prevention after thrombus resolution.

Phase 6 (Long-term secondary prevention): Given the patient’s extremely high residual cardiovascular risk, long-term dual-pathway inhibition (DPI) with aspirin (100 mg daily) plus low-dose rivaroxaban (2.5 mg twice daily) was initiated at 12 months post-PCI. The detailed phase-wise clinical context, assessment tools, objective thresholds, imaging findings, and corresponding antithrombotic adjustments are summarized in Supplementary Table S1.

### 2.6 Follow-up and outcomes

**FIGURE 4:**
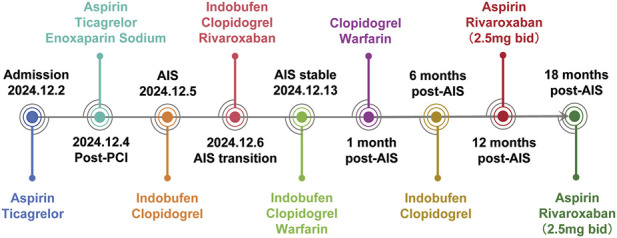
Clinical timeline. Schematic illustration of key clinical events, diagnostic findings, and the six-phase individualized antithrombotic strategy from initial presentation to 18-month follow-up. Abbreviations: PCI, percutaneous coronary intervention; AIS, acute ischemic stroke; LVT, left ventricular thrombus; DPI, dual-pathway inhibition.

During 18 months of follow-up, there were no recurrences of LVT, ischemic events (stroke, myocardial infarction, stent thrombosis) or major bleeding. The progression timeline for this case is summarized in Figure 4. Serial data on thrombus size, LVEF and laboratory markers are detailed in Supplementary Table S2.

## 3 Discussion

This case report demonstrates how an individualized, dynamic antithrombotic strategy successfully navigated a catastrophic cascade of events—from delayed reperfusion in anterior myocardial infarction to left ventricular thrombus (LVT) formation, culminating in severe cerebral embolism. Beyond successfully mitigating this triple threat, the case underscores three critical principles in managing post-infarction LVT: high-risk individuals require early, precise screening; in the absence of guideline-directed protocols, a dynamic equilibrium between thrombotic and hemorrhagic risks must be maintained; and the therapeutic horizon must extend beyond acute crisis management to encompass lifelong residual risk control.

Understanding the epidemiology and risk factors for LVT is essential for identifying patients who warrant vigilant surveillance. In the contemporary PCI era, the overall incidence of LVT following all STEMI is 2.7%, rising to 9.1% after anterior STEMI (Robinson et al., 2016). However, when assessed by cardiac magnetic resonance (CMR), detection rates following STEMI range from 6.3% to 12.2%, and reach 19.2% in anterior STEMI patients with LVEF <50% (Bulluck et al., 2018). These data suggest that the true incidence of LVT may be substantially underestimated by routine echocardiography. Regarding the optimal timing for detection, LVT incidence peaks between 9 and 12 days post-infarction (approximately 25%), whereas within the first 5 days it is below 10% (Gellen et al., 2017). This temporal pattern has important implications for surveillance strategies.

Previous studies have established the classic high-risk triad for LVT formation: anterior infarction, severe apical wall motion abnormalities or aneurysm, and LVEF <40% (Delewi et al., 2012). Additionally, diabetes mellitus and metabolic syndrome induce a prothrombotic state and pro-inflammatory response that substantially increase thrombotic risk (Chan et al., 2008). In this case, several additional factors likely contributed to the persistence of LVT: delayed revascularization (prolonging myocardial necrosis and inflammation) (Seah et al., 2025), suboptimal glycemic and lipid control (reflecting the underlying metabolic syndrome) (Mach et al., 2024), progressive left ventricular remodeling (apical aneurysm formation), and coronary artery ectasia with slow flow—a feature independently associated with heightened thrombogenic potential (Liang et al., 2019). Thus, even young patients with clustering of these features warrant early screening. Transthoracic echocardiography (TTE) remains the preferred initial investigation, though its sensitivity for detecting early or apical thrombi is limited (47%–58%) (Phuah et al., 2023). Contrast-enhanced TTE improves endocardial border definition, increasing sensitivity to approximately 64% (Weinsaft et al., 2016). CMR demonstrates superior accuracy, with sensitivity of 82%–88% in complex cases (Srichai et al., 2006), though cost and limited accessibility constrain widespread use. Notably, echocardiographically assessed apical wall motion scores may effectively identify candidates for CMR; higher scores demonstrate nearly 100% sensitivity and negative predictive value for LVT, potentially sparing over half of patients from unnecessary CMR investigation (Weinsaft et al., 2016).

The pathophysiological basis for LVT anticoagulation follows Virchow’s triad: stasis, endothelial injury, and hypercoagulability (Byrne et al., 2023). Historically, guidelines have offered Class IIb recommendations for prophylactic anticoagulation in high-risk individuals—particularly those with anterior STEMI or apical akinesis (O'Gara et al., 2013). However, Shavadia et al. demonstrated that in anterior STEMI patients with LVT, prophylactic vitamin K antagonist (VKA) use failed to reduce ischemic events but was associated with significantly higher bleeding complications (Shavadia et al., 2017). Mirroring this evidence, our patient developed LVT despite prophylactic enoxaparin after PCI.

The choice of anticoagulant for LVT must balance efficacy, safety, and individual patient characteristics. Historically, VKAs have been the cornerstone of LVT management, supported by extensive clinical experience and guidelines (Byrne et al., 2023; Kleindorfer et al., 2021; O'Gara et al., 2013). In recent years, DOAC use in LVT has increased substantially, and the 2022 AHA scientific statement endorsed DOACs as a reasonable alternative for appropriately selected patients (Levine et al., 2022). Multiple observational meta-analyses have shown comparable efficacy and safety to VKAs (Alcalai et al., 2022; Dalia et al., 2021; Youssef et al., 2023). Notably, the RIVAWAR trial—the largest randomized controlled trial to date—found no significant differences between rivaroxaban and warfarin in thrombus resolution, mortality, or bleeding at 3 months (Shah et al., 2025). From a pharmacological perspective, rivaroxaban provides rapid, predictable factor Xa inhibition (peak 1.5–2.0 h, half-life 5–9 h) (Mueck et al., 2014), making it ideal for acute post-stroke anticoagulation. This supported our initial choice of rivaroxaban. However, rivaroxaban does not affect preformed thrombin nor reduce circulating prothrombin (factor II) levels. In contrast, Warfarin exerts broader suppression of the coagulation cascade by inhibiting vitamin K-dependent synthesis of multiple factors (II, VII, IX, X). This leads to a gradual, time-dependent depletion of these factors, with prothrombin (factor II) having the longest half-life (∼60 h) (Lal et al., 2006). Such depletion may be particularly advantageous for established, fibrin-rich thrombi, as it directly reduces the substrate for new fibrin formation (Chen et al., 2018). Mechanistically, this difference could provide a theoretical rationale for switching to warfarin when early thrombus response to a single-target Xa inhibitor is suboptimal. However, in the absence of direct thrombus tissue characterization (e.g., by CMR or histopathology), this interpretation remains hypothesis-generating and speculative, warranting validation in future studies with dedicated imaging.

Despite the general effectiveness of DOACs in LVT, a subset of patients fails to respond—typically those with severe left ventricular dysfunction (LVEF ≤30–35%), apical aneurysm, metabolic syndrome, or advanced kidney disease (Alenazi et al., 2023). For example, a patient with ischemic cardiomyopathy and multiple risk factors developed recurrent stroke and LVT progression on apixaban, with complete resolution only after switching to warfarin (Farooqui et al., 2022). Conversely, treatment failure can also occur on VKAs: a case reported persistent thrombus despite 8 months of warfarin following initial dabigatran failure, highlighting that older, organized thrombi are less likely to dissolve regardless of the anticoagulant class (Fredgart and Gill, 2018). Thus, there is no uniformly superior agent; the ideal strategy must be individually tailored. A structured summary of the mechanisms, key benefits, and main risks of each antithrombotic drug used in this case is provided in Supplementary Table S3. Our experience suggests that combining dynamic risk assessment, imaging evaluation, metabolic control and guided drug switching may contribute to the successful resolution of LVT. However, robust evidence is lacking. Prospective studies are urgently needed to define early failure criteria and optimal switching strategies, especially in ultra-high-risk patients with multiple prothrombotic features.

Current guidelines recommend anticoagulation therapy for 3–6 months in patients with LVT (Ibanez et al., 2018; O'Gara et al., 2013). However, management becomes particularly complex when LVT is complicated by recent PCI and stroke, as these patients face both high thrombotic and bleeding risks. To mitigate bleeding, we used indobufen (a reversible COX-1 inhibitor) instead of aspirin during the acute stroke phase. The OPTION trial showed a 37% bleeding reduction with indobufen versus aspirin (HR: 0.63) without increased ischemic events (Wu et al., 2023), and the ASPIRATION registry confirmed even lower bleeding (HR: 0.24) with similar MACE (HR: 0.99) (Dai et al., 2024). Regarding anticoagulation resumption after cardioembolic stroke, early initiation (≤4 days) is supported by two large meta-analyses (5,441 and 17,380 patients), showing reduced recurrent stroke without increasing symptomatic intracranial hemorrhage (Bafail and Bafail, 2025; Dehbi et al., 2025). In our patient, NIHSS improved from 8 to 3 by day 2, with no hemorrhage on CT and stable bleeding markers; accordingly, anticoagulation was resumed on day 2, consistent with this evidence. Currently, direct evidence regarding the choice between dual and triple antithrombotic therapy in patients with LVT undergoing PCI is lacking; therefore, antithrombotic strategies have been guided by evidence from AF-PCI populations. Ischemic risk peaks within the first month post-PCI and then declines steeply, whereas bleeding risk reaches a stable plateau after an initial periprocedural rise (Greco et al., 2026). This temporal dissociation supports early intensive therapy followed by later de-escalation. Accordingly, the 2023 ESC ACS and 2024 ESC AF guidelines recommend a short course of triple therapy (OAC + DAPT) for 1 week (Class I) or up to 30 days (Class IIa) in patients with acceptable bleeding risk, followed by de-escalation to dual therapy (OAC + single antiplatelet) (Byrne et al., 2023; Van Gelder et al., 2024). Evidence from landmark AF-PCI trials (AUGUSTUS, RE-DUAL PCI) and network meta-analyses has consistently shown that DOAC-based dual therapy significantly reduces major bleeding compared with VKA-based triple therapy, without increasing stent thrombosis or MACE (Cannon et al., 2017; Lopes et al., 2019; Lopes et al., 2020). Real-world data confirmed the bleeding reduction but noted a modest increase in stent thrombosis with dual therapy compared with triple therapy (Park et al., 2025). In our case, after 1 month of triple therapy (warfarin + DAPT, INR target 2.0–2.5) and complete revascularization, we de-escalated to dual therapy (warfarin + clopidogrel). Importantly, neither dual nor triple antithrombotic therapy represents a one-size-fits-all solution; the optimal strategy must be dynamically adjusted, with interventions stratified according to evolving risk, rather than mechanically adhering to a fixed, long-term regimen.

Crucially, LVT resolution does not eliminate cardiovascular risk. The reported recurrence rate of LVT ranges from 11.2% to 24.3% (Katsouras et al., 2025). Our patient’s residual risk (metabolic syndrome, aneurysm, ectasia, prior thromboembolism) remains high, requiring lifelong secondary prevention. Dual pathway inhibition (DPI) with aspirin + low-dose rivaroxaban synergistically targets platelet aggregation and thrombin generation. The COMPASS trial demonstrated a 24% reduction in cardiovascular death, stroke, or MI and an 18% reduction in all-cause mortality versus aspirin alone, without a significant increase in fatal bleeding (Eikelboom et al., 2017). Current guidelines recommend DPI for high-risk patients with chronic coronary disease (Class IIa, Level B) (Virani et al., 2023). Given the patient’s persistently high-risk profile, we implemented DPI as a long-term secondary prevention strategy.

As a single-case study, findings are not generalizable. Limitations include: the CMR or histopathological examination was not performed to characterize thrombus tissue because the patient could not cooperate; the 18-month follow-up is relatively short, precluding definitive conclusions about lifelong efficacy; and the decision to switch from rivaroxaban to warfarin was based on echocardiographic findings and clinical judgment rather than a validated protocol. Nevertheless, this report provides a detailed real-world example of dynamic risk-adapted antithrombotic management in a complex, poorly evidenced scenario.

## 4 Conclusion

This case report highlights the importance of an individualized, phased antithrombotic strategy for managing the complex clinical dilemma of left ventricular thrombus and acute stroke following myocardial infarction. Serial dynamic risk assessments and imaging evaluations help balance the dual threats of ischemia and hemorrhage and guide appropriate treatment adjustments. Although the findings provide valuable clinical insights into the management of cardiovascular-cerebrovascular overlap syndromes for which evidence-based guidelines are lacking, they are derived from a single case. Prospective studies are required to optimize the timing and criteria for drug switching and to validate this approach in larger populations.

## Data Availability

The original contributions presented in the study are included in the article/Supplementary Material, further inquiries can be directed to the corresponding authors.
