# Supplementary material for: From heart to brain: a Case Report of individualized antithrombotic management for left ventricular thrombus and stroke in a young patient with acute myocardial infarction
Source: Front Pharmacol. 2026 Jun 11;17:1829968. doi: 10.3389/fphar.2026.1829968 (PMC13293799; doi:10.3389/fphar.2026.1829968)
Supplement: Supplementary file 1 [file Table1.docx]

**Supplementary Table S1.** Six-phase antithrombotic strategy overview.

| Phase | Timing / clinical context | Key risk assessment (thresholds) | Imaging findings | Antithrombotic regimen |
| --- | --- | --- | --- | --- |
| 0 | Post‑PCI, pre‑stroke | GRACE 127;  CRUSADE 30; | Apical blood stasis | Aspirin  ticagrelor  enoxaparin |
| 1 | Acute ischemic stroke (day 1 post‑PCI) | NIHSS 8;  SITS‑SICH 8 | Occipital infarct (3.5×3.1 cm) | Indobufen clopidogrel |
| 2 | Neurological improvement (day 2) | NIHSS 8→3; SITS‑SICH 8→4;  CT no bleed;  Hb stable | Mobile LVT (1.6×1.1 cm, 1.3×1.1 cm) | Indobufen  clopidogrel  rivaroxaban |
| 3 | Early reassessment (day 7) | CRUSADE 30→20;  HAS‑BLED 3→2; | LVT unchanged | Indobufen clopidogrel  warfarin  (INR 2.0–2.5) |
| 4 | Complete revascularization (1 month) | Reduced thrombus size/mobility;  RCA‑PCI success | Apical aneurysm (3.0×1.5 cm)； residual LVT (1.8×0.7 cm) | Warfarin clopidogrel |
| 5 | LVT resolution (6 months) | Contrast TTE: complete LVT resolution | No thrombus | Indobufen clopidogrel |
| 6 | Long‑term prevention (12 months onward) | High residual risk | No recurrence | Aspirin rivaroxaban (2.5 mg bid ) |

**Abbreviations:** PCI, percutaneous coronary intervention; TTE, transthoracic echocardiography; NIHSS, National Institutes of Health Stroke Scale; SITS‑SICH, Safe Implementation of Thrombolysis in Stroke-Symptomatic Intracerebral Hemorrhage; Hb, hemoglobin; LVT, left ventricular thrombus; HAS‑BLED, Hypertension, Abnormal renal/liver function, Stroke, Bleeding, Labile INR, Elderly, Drugs/alcohol; CRUSADE (Can Rapid risk stratification of Unstable angina patients Suppress ADverse outcomes with Early implementation of the ACC/AHA guidelines) — a bleeding risk score for patients undergoing PCI, with score ranges: ≤20 (very low risk, 3.1%), 21–30 (low risk, 5.5%), 31–40 (moderate risk, 8.6%), 41–50 (high risk, 11.9%), >50 (very high risk, 19.5%) ; INR, international normalized ratio; RCA, right coronary artery.
