# Supplementary material for: From heart to brain: a Case Report of individualized antithrombotic management for left ventricular thrombus and stroke in a young patient with acute myocardial infarction
Source: Front Pharmacol. 2026 Jun 11;17:1829968. doi: 10.3389/fphar.2026.1829968 (PMC13293799; doi:10.3389/fphar.2026.1829968)
Supplement: Supplementary file 2 [file Table2.docx]

**Supplementary Table S2.** Serial changes in LVEF, thrombus size, and laboratory markers during follow-up.

| Parameters | Baseline (pre‑PCI) | Day 1 (post‑stroke) | Day 7 (Switch to warfarin) | 1 month (post‑revascularization) | 6 months (LVT resolved) | 18 months |
| --- | --- | --- | --- | --- | --- | --- |
| Thrombus size (cm) | None | 1.6x1.1&1.3x1.1 | 1.6x1.2&1.3x1.1 | 1.8x0.7 | Complete resolution | No recurrence |
| LVEF (%) | 46 | 32 | 41 | 40 | 44 | 52 |
| NT‑proBNP (ng/L) | 2226 | 1580 | 1120 | 1453 | 632 | 239 |
| cTnI (ug/L) | 1.7 | 1.1 | 0.069 | 0.08 | — | — |
| D‑dimer (mg/L) | 0.97 | 1.01 | 0.32 | 0.06 | — | — |
| PT (s) | 11.7 | 12.1 | 12.9 | 27.7 | 22.1 | — |
| APTT (s) | 29.1 | 29.9 | 32.3 | 47.2 | 47.1 | — |
| CRP(mg/L) | 55.36 | 93.83 | 40.29 | 13.41 | 8.31 | — |

**Abbreviations:** LVEF, left ventricular ejection fraction; PCI, percutaneous coronary intervention; LVT, left ventricular thrombus; PT, prothrombin time; APTT, activated partial thromboplastin time; INR, international normalized ratio; cTnI, cardiac troponin I; NT-proBNP, N-terminal pro-B-type natriuretic peptide; CRP, C-reactive protein. “—” indicates not measured or not applicable..
