# Supplementary material for: From heart to brain: a Case Report of individualized antithrombotic management for left ventricular thrombus and stroke in a young patient with acute myocardial infarction
Source: Front Pharmacol. 2026 Jun 11;17:1829968. doi: 10.3389/fphar.2026.1829968 (PMC13293799; doi:10.3389/fphar.2026.1829968)
Supplement: Supplementary file 3 [file Table3.docx]

**Supplementary Table S3.** Risk-benefit profile of antithrombotic drugs used in the case.

| Drug (phase) | Mechanism of action | Key benefits | Main risks / adverse effects |
| --- | --- | --- | --- |
| Aspirin (0, 6) | Irreversible COX‑1 inhibition; suppresses thromboxane A₂ synthesis. | Standard antiplatelet for secondary prevention; low cost; synergistic with low‑dose rivaroxaban in DPI. | Gastrointestinal injury; bleeding tendency; allergic reactions; salicylate reactions. |
| Ticagrelor (0) | Reversible P2Y₁₂ receptor antagonist (no metabolic activation needed) ; also increases adenosine via ENT‑1 inhibition. | Rapid onset and potent inhibition; Superior ischemic reduction vs. clopidogrel; consistent antiplatelet effect regardless of CYP2C19 genotype. | Dyspnea; Increased risk of bleeding more than clopidogrel; bradycardia. |
| Enoxaparin (0) | Binds to antithrombin III, accelerating the inactivation of factors Xa and IIa | predictable dose-response; no routine monitoring required; lower bleeding than unfractionated heparin.;better prothrombin suppression. | Injection site hematoma; heparin-induced thrombocytopenia (rare); dose-dependent bleeding; |
| Indobufen (1,2,3,5) | Reversible, non‑competitive COX‑1 inhibitor; short half-life (6-8 h) | Equivalent ischemic protection; significantly lower bleeding risk; rapid reversal (<24 h) and better GI tolerability. | Less robust evidence than aspirin; Limited availability (mainly China and Italy). |
| Clopidogrel (1‑6) | Irreversible P2Y₁₂ prodrug; requires CYP2C19 activation. | Effective P2Y₁₂ inhibition across multiple phases; synergistic with oral anticoagulants; well‑established safety. | Variable antiplatelet response(CYP2C19 LoF alleles); bleeding (especially with aspirin); rare thrombotic thrombocytopenic purpura. |
| Rivaroxaban (2) | Direct, reversible factor Xa inhibitor; inhibits free and clot‑bound Xa, reducing thrombin generation. | Rapid onset (peak 1.5-2 h); predictable pharmacokinetics (half‑life 5-9 h); lower intracranial bleeding vs. Warfarin. | Anticoagulant effect cannot be routinely monitored; no effect on pre‑formed thrombin or prothrombin; GI bleeding; limited specific antagonist. |
| Warfarin (3,4) | VKORC1 inhibitor; depletes reduced vitamin K, impairing γ‑carboxylation of factors II, VII, IX, and X. | Multi‑target inhibition; INR‑guided titration; effective for fibrin‑rich, organized thrombi; reduces prothrombin (half‑life ~60 h). | Narrow therapeutic window; drug‑food interactions; delayed onset (~5 days); frequent INR monitoring required; bleeding (especially intracranial); genetic variability (CYP2C9, VKORC1) affects dosing. |

**Abbreviations:** COX‑1, cyclooxygenase‑1; DPI, dual‑pathway inhibition; ENT‑1, equilibrative nucleoside transporter‑1; GI, gastrointestinal; INR, international normalized ratio; LoF, loss‑of‑function; VKORC1, vitamin K epoxide reductase complex subunit 1.
